# Supplementary material for: Dynamics of anti-SARS-CoV-2 seroconversion in individual patients and at the population level
Source: PLoS One. 2022 Sep 9;17(9):e0274095. doi: 10.1371/journal.pone.0274095 (PMC9462561; doi:10.1371/journal.pone.0274095)
Supplement: S1 Fig — Antibodies were identified in blood samples after separation of blood sera by clotting and centrifugation. Microblot-array testing was applied to determine IgG level in each participants and positive/negative result was qualified according to the validated test. (PDF) [file pone.0274095.s001.pdf]

| | | |
|---|---|---|
| **Group 1**<br>anti-NCP IgG negative in May,<br>non-vaccinated till December | 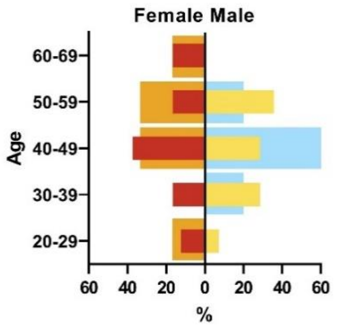 | |
| **Group 2**<br>anti-NCP IgG negative in May<br>vaccinated May-December | 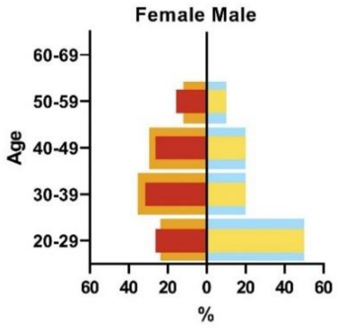 | |
| **Group 3**<br>anti-NCP IgG positive in May<br>non-vaccinated till December | 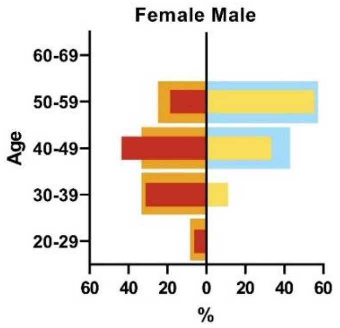 | |
| **Group 4**<br>anti-NCP IgG positive in May<br>vaccinated May-December | 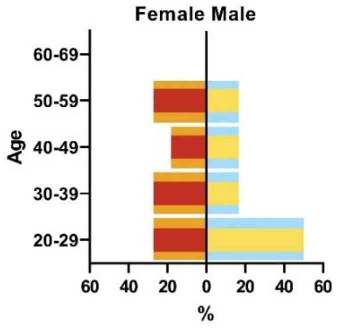 | demographic parameters of each investigated group (backgraound)<br><br>demographic parameters of the sub-population that was found immune to SARS-CoV-2 (IgG positive) |

**S1 Fig. Demographics in the serological screening of population without registered SARS-CoV-2 infections: demographic parameters of participants found positive for anti-SARS-CoV-2 IgG in each investigated group compared to general demographic parameters of the group.** Antibodies were identified in blood samples after separation of blood sera by clotting and centrifugation. Microblot-array testing was applied to determine IgG level in each participants and positive/negative result was qualified according to the validated test.
