# Supplementary material for: Dynamics of anti-SARS-CoV-2 seroconversion in individual patients and at the population level
Source: PLoS One. 2022 Sep 9;17(9):e0274095. doi: 10.1371/journal.pone.0274095 (PMC9462561; doi:10.1371/journal.pone.0274095)
Supplement: S2 Fig — (PDF) [file pone.0274095.s002.pdf]

**Group 1**<br>anti-NCP IgG negative in May,<br>non-vaccinated till December	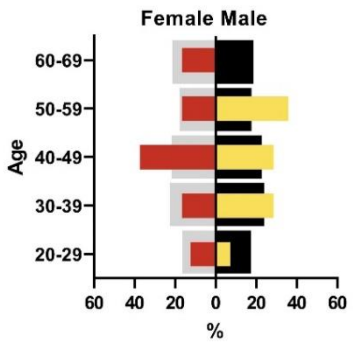	
**Group 2**<br>anti-NCP IgG negative in May<br>vaccinated May-December	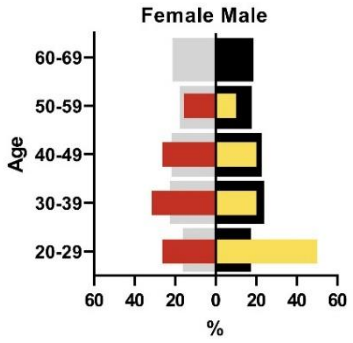	
**Group 3**<br>anti-NCP IgG positive in May<br>non-vaccinated till December	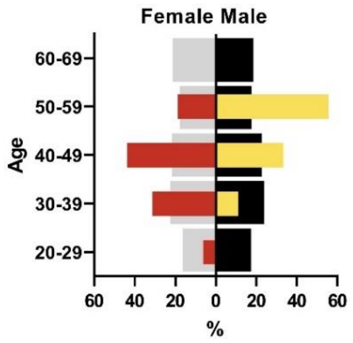	
**Group 4**<br>anti-NCP IgG positive in May<br>vaccinated May-December	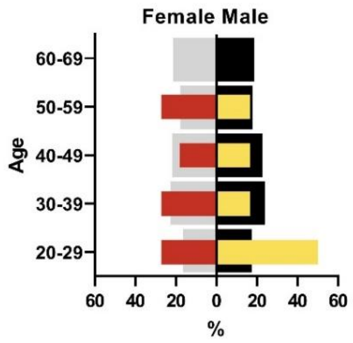	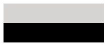<br>demographic parameters of the Polish population (whole country, background)<br>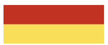<br>demographic parameters of the investigated groups

S2 Fig. Demographics in the serological screening of population without registered SARS-CoV-2 infections: demographic parameters of each investigated group compared to general demographic parameters of the whole country.
