# Supplementary material for: Dynamics of anti-SARS-CoV-2 seroconversion in individual patients and at the population level
Source: PLoS One. 2022 Sep 9;17(9):e0274095. doi: 10.1371/journal.pone.0274095 (PMC9462561; doi:10.1371/journal.pone.0274095)
Supplement: S3 Fig — (PDF) [file pone.0274095.s003.pdf]

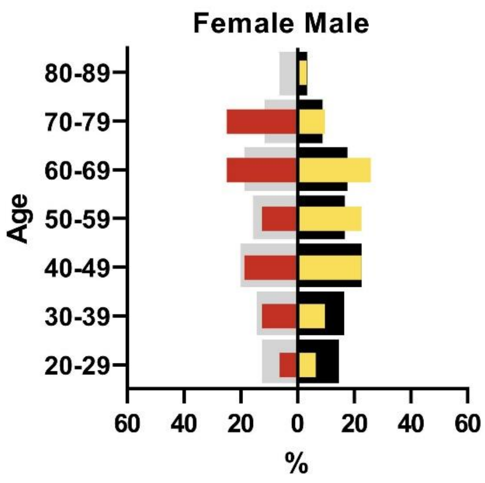

demographic parameters of the Patients hospitalized due to COVID-19

demographic parameters of the Polish population (general)

S3 Fig. Demographics in patients hospitalized due to COVID-19
