# Supplementary material for: Dynamics of anti-SARS-CoV-2 seroconversion in individual patients and at the population level
Source: PLoS One. 2022 Sep 9;17(9):e0274095. doi: 10.1371/journal.pone.0274095 (PMC9462561; doi:10.1371/journal.pone.0274095)
Supplement: S4 Fig — Anti-NCP- antibodies specific to nucleocapsid protein N, anti-RBD- antibodies specific to receptor binding domain within spike protein S1 region. Antibodies were identified in blood samples after separation of blood sera by clotting and centrifugation. Microblot-array testing was applied to determine IgG level (U/ml). Median values are presented (dash), with quartiles 1 and 3 (boxes), and minimum/maximum values (whiskers) after outlier elimination (Tukey method), dots represent outliers; p>0.05 by Welsh test. (PDF) [file pone.0274095.s004.pdf]

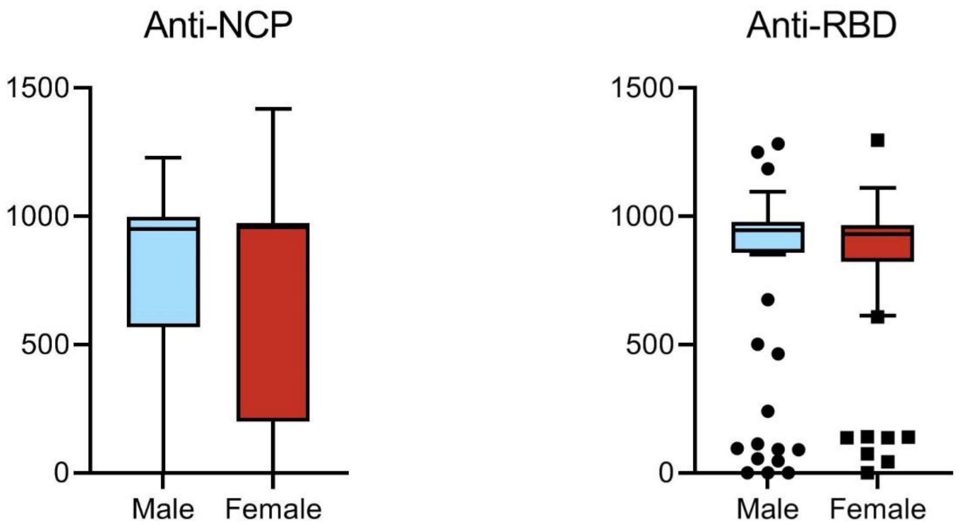

S4 Fig. **IgG specific to SARS-CoV-2 in non-vaccinated patients hospitalized due to COVID-19: female and male comparison.** Anti-NCP- antibodies specific to nucleocapsid protein N, anti-RBD- antibodies specific to receptor binding domain within spike protein S1 region. Antibodies were identified in blood samples after separation of blood sera by clotting and centrifugation. Microblot-array testing was applied to determine IgG level (U/ml). Median values are presented (dash), with quartiles 1 and 3 (boxes), and minimum/maximum values (whiskers) after outlier elimination (Tukey method), dots represent outliers; $p>0.05$ by Welsh test.
