# Supplementary material for: Dynamics of anti-SARS-CoV-2 seroconversion in individual patients and at the population level
Source: PLoS One. 2022 Sep 9;17(9):e0274095. doi: 10.1371/journal.pone.0274095 (PMC9462561; doi:10.1371/journal.pone.0274095)
Supplement: S1 Table — NCP–nucleocapsid protein, RBD–receptor binding protein, Min–minimal value, Max–maximum value, Q25%–lower quartile, Q75%–upper quartile. (PDF) [file pone.0274095.s005.pdf]

S1 Table. Distributions of quantitative variables of measurements for IgG specific to SARS-CoV-2 NCP and RBD in patients: Group 1: anti-NCP IgG negative and non-vaccinated patients ; Group 2: anti-NCP IgG negative and vaccinated patients; Group 3: anti-NCP IgG positive and non-vaccinated patients; Group 4: anti-NCP IgG positive and vaccinated patients. NCP – nucleocapsid protein, RBD – receptor binding protein, Min – minimal value, Max – maximum value, Q25% – lower quartile, Q75% – upper quartile.

Group	Antibody	Date of the study	N	Min	Q25%	Median	Q75%	Max	Range	Average
1	NCP	May	38	0	0	5.211	17.27	107.3	107.3	13.11
		December	38	0	13.82	20.96	416.5	986.2	986.2	219
	RBD	May	38	0	0	0.691	6.769	120	120	6.172
		December	38	19.77	38.09	58.61	278.8	976.7	956.9	207.6
2	NCP	May	29	0	0.0605	8.425	39.6	170.7	170.7	27.53
		December	29	5.701	14.55	21.6	26.16	400	394.3	51.78
	RBD	May	29	0	0	0.398	7.759	64.88	64.88	7.461
		December	29	59.99	457.1	809.6	943.7	987.2	927.2	696.6
3	NCP	May	25	317	752	929	989.9	1343	1026	844.6
		December	25	0	41.91	158.6	265.1	818.9	818.9	196.7
	RBD	May	25	11.73	305.2	922.5	982.9	1221	1209	683.2
		December	25	5.202	112	202.7	492.1	935.5	930.3	308.9
4	NCP	May	17	238.1	553.9	862.1	969.6	1279	1041	774
		December	17	19.61	35.1	91.53	133.6	476.9	457.2	118.8
	RBD	May	17	0	220.4	558.4	805.3	1221	1221	518.5
		December	17	461.5	759	941.6	972.9	988.5	527	862
