# Supplementary material for: Dynamics of anti-SARS-CoV-2 seroconversion in individual patients and at the population level
Source: PLoS One. 2022 Sep 9;17(9):e0274095. doi: 10.1371/journal.pone.0274095 (PMC9462561; doi:10.1371/journal.pone.0274095)
Supplement: S2 Table — D5-D90 –days from 5 to 90 representing estimated number of days after onset of infection; NCP–nucleocapsid protein, RBD–receptor binding protein, Min–minimal value, Max–maximum value, Q25%–lower quartile, Q75%–upper quartile. (PDF) [file pone.0274095.s006.pdf]

S2 Table. Distributions of quantitative variables of measurements for IgG specific to SARS-CoV-2 NCP and RBD in groups of hospitalized patients in selected days. D5-D90 – days from 5 to 90 representing estimated number of days after onset of infection; NCP – nucleocapsid protein, RBD – receptor binding protein, Min – minimal value, Max – maximum value, Q25% – lower quartile, Q75% – upper quartile.

		N	Min	Q25%	Median	Q75%	Max	Average
non-vaccinated	NP	95	0	469.3	957.1	988	1418	755.1
	RBD	95	0	830	940.4	972.5	1297	790.9
male non-vaccinated	NP	56	0	567	950.3	999.5	1229	776.3
	RBD	56	0	858.5	946	978.9	1283	797.1
female non-vaccinated	NP	40	0	200.9	957.7	974.5	1418	729.8
	RBD	40	0	823.2	932.1	965.2	1297	786.7
Mild anti-NCP	D5	5	0	4.527	42.87	565.9	944.1	236.7
	D10	11	0	3.067	483.3	957.2	964	474.6
	D15	17	16.64	189.1	942.9	971.5	1229	657.8
	D30	6	773.3	786	918.8	969.2	1002	893.5
	D90	2	988	988	988.1	988.1	988.1	988.1
Moderate anti-NCP	D10	4	850.4	877.3	961.3	967.7	968.7	935.4
	D15	12	469.3	972.8	980.8	1018	1418	994.9
	D30	8	950.2	974.2	1002	1057	1182	1022
	D90	7	817.9	950.5	966.4	988.5	988.7	952.1
Severe anti-NCP	D10	5	0	0	58.18	693.2	945.1	288.9
	D15	7	33.99	65.59	841.3	1066	1102	698.2
	D30	4	1002	1004	1059	1145	1157	1069
	D90	4	868.8	881.4	941.1	981.2	987.2	934.6
Mild anti-RBD	D5	5	47.38	69.21	140.2	927.1	944.1	426.6
	D10	11	0	96.13	501.9	957.2	964	516.9
	D15	17	0	610.1	930.1	952.2	1283	771.3
	D30	6	883.7	903.9	940.4	988.6	1004	943.8
	D90	2	970.6	970.6	973.8	976.9	976.9	973.8
Moderate anti-RBD	D10	4	830	835.1	878.3	954.1	970.1	889.2
	D15	12	675.2	916.8	963.9	1015	1297	966.7
	D30	8	882.9	932.2	1005	1056	1112	999.8
	D90	7	915.3	944.6	959	977.6	988.7	957.9
Severe Anti-RBD	D10	5	0	27.71	138.9	718.7	972.5	326.3
	D15	7	0	92.36	936.4	1186	1250	760.6
	D30	4	1002	1002	1013	1078	1096	1031
	D90	4	906.8	909.3	940.2	967.3	968.6	938.9
