# Supplementary material for: Dynamics of anti-SARS-CoV-2 seroconversion in individual patients and at the population level
Source: PLoS One. 2022 Sep 9;17(9):e0274095. doi: 10.1371/journal.pone.0274095 (PMC9462561; doi:10.1371/journal.pone.0274095)
Supplement: S3 Table — (PDF) [file pone.0274095.s007.pdf]

S3 Table. Number and percentage of smokers and allergies in studied groups.

	Group 1 (N=38) negative for anti-NCP IgG in May. non-vaccinated	Group 2 (N=29) negative for anti-NCP IgG in May. vaccinated	Group 3 (N=25) positive for anti-NCP IgG in May. non-vaccinated	Group 4 (N=17) positive for anti-NCP IgG in May. vaccinated
Allergy	11 (29%)	7 (24%)	4 (16%)	6 (35%)
Smokers	11 (29%)	7 (24%)	8 (32%)	3 (18%)
