# Supplementary material for: Dynamics of anti-SARS-CoV-2 seroconversion in individual patients and at the population level
Source: PLoS One. 2022 Sep 9;17(9):e0274095. doi: 10.1371/journal.pone.0274095 (PMC9462561; doi:10.1371/journal.pone.0274095)
Supplement: S4 Table — (PDF) [file pone.0274095.s008.pdf]

S4 Table. Distribution of BMI index in the study groups								
	Min	Q1	Median	Q3	Max	Mean	Std. Deviation	Std. Error of Mean
Group 1 (N=38) negative for anti-NCP IgG in May. non-vaccinated	19.05	21.92	25.57	29.36	35.32	25.84	4.58	0.74
Group 2 (N=29) negative for anti-NCP IgG in May. vaccinated	17.1	21.81	23.83	28.36	33.79	24.79	4.56	0.85
Group 3 (N=25) positive for anti-NCP IgG in May. non-vaccinated	20.29	22.81	27.77	30.2	35.16	27.05	4.59	0.92
Group 4 (N=17) positive for anti-NCP IgG in May. vaccinated	17.36	20.24	23.53	28.42	38.58	24.74	5.53	1.34
Q25% and Q75% - 1. and 3. Quantile								
