# Supplementary material for: Dynamics of anti-SARS-CoV-2 seroconversion in individual patients and at the population level
Source: PLoS One. 2022 Sep 9;17(9):e0274095. doi: 10.1371/journal.pone.0274095 (PMC9462561; doi:10.1371/journal.pone.0274095)
Supplement: S5 Table — D5-D90 –days from 5 to 90 representing estimated number of days after onset of infection, t-test was used, p-values (adjusted) are presented. (PDF) [file pone.0274095.s009.pdf]

**S5 Table. Statistical analysis of differences in average anti-RBD IgG levels detected in groups of hospitalized patients in selected days.** D5-D90 – days from 5 to 90 representing estimated number of days after onset of infection, t-test was used, p-values (adjusted) are presented

| Anti-RBD | | | | | |
|---|---|---|---|---|---|
| Mild | D5 | D10 | D15 | D30 | D90 |
| D5 | - | > 0.1 | > 0.1 | > 0.1 | > 0.1 |
| D10 | > 0.1 | - | **< 0.01** | **< 0.05** | **< 0.05** |
| D15 | > 0.1 | **< 0.01** | - | > 0.1 | > 0.1 |
| D30 | > 0.1 | **< 0.05** | > 0.1 | - | > 0.1 |
| D90 | > 0.1 | **< 0.05** | > 0.1 | > 0.1 | - |
| | | | | | |
| Moderate | D5 | D10 | D15 | D30 | D90 |
| D5 | - | - | - | - | - |
| D10 | - | - | **< 0.001** | **< 0.05** | > 0.1 |
| D15 | - | **< 0.001** | - | > 0.1 | > 0.1 |
| D30 | - | **< 0.05** | > 0.1 | - | > 0.1 |
| D90 | - | > 0.1 | > 0.1 | > 0.1 | - |
| | | | | | |
| Severe | D5 | D10 | D15 | D30 | D90 |
| D10 | > 0.1 | - | > 0.1 | **< 0.05** | > 0.1 |
| D15 | > 0.1 | > 0.1 | - | > 0.1 | > 0.1 |
| D30 | > 0.1 | **< 0.05** | > 0.1 | - | **0.1 > p > 0.05** |
| D90 | > 0.1 | > 0.1 | > 0.1 | **0.1 > p > 0.05** | - |
