# Supplementary material for: Dynamics of anti-SARS-CoV-2 seroconversion in individual patients and at the population level
Source: PLoS One. 2022 Sep 9;17(9):e0274095. doi: 10.1371/journal.pone.0274095 (PMC9462561; doi:10.1371/journal.pone.0274095)
Supplement: S6 Table — D5-D90 –days from 5 to 90 representing estimated number of days after onset of infection, t-test was used, p-values (adjusted) are presented. (PDF) [file pone.0274095.s010.pdf]

S6 Table. Statistical analysis of differences in average anti-NCP IgG levels detected in groups of hospitalized patients in selected days. D5-D90 – days from 5 to 90 representing estimated number of days after onset of infection, t-test was used, p-values (adjusted) are presented

Anti -NCP					
Mild	D5	D10	D15	D30	D90
D5	-	> 0.1	> 0.1	**0.1 > p > 0.05**	> 0.1
D10	> 0.1	-	> 0.1	**0.1 > p > 0.05**	**0.1 > p > 0.05**
D15	> 0.1	> 0.1	-	> 0.1	> 0.1
D30	**0.1 > p > 0.05**	**0.1 > p > 0.05**	> 0.1	-	> 0.1
D90	> 0.1	**0.1 > p > 0.05**	> 0.1	> 0.1	-
Moderate	D5	D10	D15	D30	D90
D5	-	-	-	-	-
D10	-	-	> 0.1	> 0.1	> 0.1
D15	-	> 0.1	-	> 0.1	> 0.1
D30	-	> 0.1	> 0.1	-	**< 0.05**
D90	-	> 0.1	> 0.1	**< 0.05**	**-**
Severe	D5	D10	D15	D30	D90
D10	**0.1 > p > 0.05**	-	> 0.1	**0.1 > p > 0.05**	> 0.1
D15	> 0.1	> 0.1	-	> 0.1	> 0.1
D30	> 0.1	**0.1 > p > 0.05**	> 0.1	-	**< 0.05**
D90	> 0.1	> 0.1	> 0.1	**< 0.05**	-
